# A Systematic Review of Electrophysiological Findings in Binge-Purge Eating Disorders: A Window Into Brain Dynamics

**DOI:** 10.3389/fpsyg.2021.619780

**Published:** 2021-04-29

**Authors:** Joao C. Hiluy, Isabel A. David, Adriana F. C. Daquer, Monica Duchesne, Eliane Volchan, Jose C. Appolinario

**Affiliations:** ^1^Obesity and Eating Disorders Group, Institute of Psychiatry, Federal University of Rio de Janeiro, Rio de Janeiro, Brazil; ^2^Laboratory of Behavioral Neurophysiology, Physiology and Pharmacology Department, Biomedical Institute, Federal Fluminense University, Niteroi, Brazil; ^3^State Institute of Diabetes and Endocrinology, Rio de Janeiro, Brazil; ^4^Carlos Chagas Filho Institute of Biophysics, Federal University of Rio de Janeiro, Rio de Janeiro, Brazil

**Keywords:** EEG, binge eating, neurophysiology, bulimia nervosa, electroneurophysiology, ERP, frequency analysis

## Abstract

Binge-purge eating disorders (BP-ED), such as bulimia nervosa and binge eating disorder, may share some neurobiological features. Electroencephalography (EEG) is a non-invasive measurement modality that may aid in research and diagnosis of BP-ED. We conducted a systematic review of the literature on EEG findings in BP-ED, seeking to summarize and analyze the current evidence, as well as identify shortcomings and gaps to inform new perspectives for future studies. Following PRISMA Statement recommendations, the PubMed, Embase, and Web of Science databases were searched using terms related to “electroencephalography” and “binge-purge” eating disorders. Of 555 articles retrieved, 15 met predefined inclusion criteria and were included for full-text analysis. Eleven studies investigated EEG by means of event-related potentials (ERP) in BP-ED individuals: 7 using eating disorder-related stimuli (i.e., food, body image) and 4 using non-eating disorder-related stimuli (i.e., facial expressions or auditory clicks). These studies found significant differences in the N200, P200, P300, and LPP components in BP-ED participants compared to controls, indicating that this population exhibits impairments in selective attention, attentional allocation/processing, and allocation of motivational or emotion-based attention. Five studies investigated EEG using frequency analysis; reporting significant differences in beta activity in fronto-temporal and occipito-temporo-parietal areas in BP-ED individuals compared to controls, revealing a dysfunctional brain network. However, the small number of studies, the heterogeneity of samples, study paradigms, stimulus types, and the lack of an adequate assessment of neuropsychological parameters are some limitations of the current literature. Although some EEG data are promising and consistent with neuroimaging and neuropsychological findings in individuals with BP-ED, future studies need to overcome current methodological shortcomings.

## Introduction

Eating disorders are characterized by a persistent disturbance of eating or eating-related behavior that results in the altered consumption or absorption of food, with a significant impact on physical health or psychosocial functioning (American Psychiatric Association, 2013). Although the eating disorders field has witnessed significant development in the last decades, there is scant information about the pathophysiology of these conditions. A better understanding of the neurophysiology of eating disorders is crucial for improvement in diagnosis and treatment, and still represents a great challenge for clinicians and researchers (Fairburn and Harrison, 2003; Jansen, 2016). Studies of neuroimaging, neuropsychological assessments, and genetic investigation have been performed to elucidate the pathophysiology of EDs. In line with the recommendations of the Research Domain Criteria framework (Insel et al., 2010; U. S. Department of Health Human Services, 2020), some research has begun to look for possible neurobiological markers of eating-disordered behaviors.

According to the main diagnostic classifications (the Diagnostic and Statistical Manual of Mental Disorders, DSM, and the International Classification of Diseases, ICD), the ED group is divided into several categories, with anorexia nervosa (AN), bulimia nervosa (BN), and binge eating disorder (BED) as the three major diagnostic rubrics. However, there is a growing body of evidence suggesting an alternative, dimensional classification based on distinct clinical and neurobiological characteristics (Brooks et al., 2012). Specifically, authors hypothesized that ED could be described as a continuum, with disorders characterized by restriction and appetite control (AN) on one pole and, at the opposite pole, disorders characterized by loss of control and increased appetite (BN and BED) (Fairburn and Harrison, 2003). The restricted-type EDs are associated with such cognitive disturbances as perfectionism, cognitive rigidity, problems with change of focus and excessive attention to detail, ruminations, and obsessions about feeding and worries with body weight (Tchanturia et al., 2002, 2004; Startup et al., 2013; Kułakowska et al., 2014). On the other hand, the impulsive-type EDs (which include BN and BED) are characterized by impulsiveness, high excitability, borderline personality traits, and a higher rate of substance abuse (Verkes et al., 1996; Krug et al., 2009; Monica et al., 2010; Pearson et al., 2017).

Some neurobiological findings have been described in this subgroup of binge-purge eating disorders (BP-ED). For instance, functional magnetic resonance imaging (fMRI) studies have shown that individuals with BED exhibit increased activity in the medial orbitofrontal cortex, a reward-processing area of the brain, in response to images of high-calorie foods (Schienle et al., 2009). Additionally, other neuroimaging methods, as well as neuropsychological assessments, have suggested that BP-ED is associated with altered function in cortical and striatal regions, increased attentional shifting to foods, an increase in aspects related to the desire for food, and a decreased sensitivity to reward (Kessler et al., 2016).

Current evidence suggests that the attention domain is particularly relevant in the cognitive processes of individuals with BP-ED; individuals in this subgroup exhibit specific attentional bias (Stojek et al., 2018). For example, in 2016, Albery et al. used an emotional-Stroop task to measure attentional biases in individuals with BN (Albery et al., 2016). They found a cognitive bias for food and body-related cues in individuals with BN, but not in controls. Likewise, Leehr et al. compared the valuation of food vs. non-food stimuli by overweight subjects with BED, overweight subjects without BED, and normal-weight controls and found that the BED group showed a significantly stronger food bias compared to non-BED overweight subjects (Leehr et al., 2016). Preliminary evidence suggests that attentional bias to disorder-specific stimulus (such as food or weigh/shape cues) may be a potent contributor to the development and maintenance of binge eating.

An interesting method to assess the underlying brain physiology is electroencephalography (EEG), a low-cost technique with excellent temporal resolution that consists of recording the electrical activity produced by cortical neurons in the brain through electrodes placed on the scalp (Niedermeyer and da Silva, 2004). Several analyses can be applied to the EEG signal, of which two are commonly used for studying neuroelectrophysiological parameters: time domain analysis and frequency domain analysis.

Time domain analysis (event-related potential, ERP) assesses the systematic positive or negative voltage deflections (electrical brain waves) that occur in response to repeated stimuli or motor events (Luck, 2014; Kessler et al., 2016). Study of ERPs allows recording of the time course of cortical processes of related events in a time-locked manner with a specific stimulus. ERPs are typically distinguished by their time window of occurrence, morphology, topography on the scalp, and response to experimental manipulations (Luck, 2014). ERP waveforms may be associated with motor, cognitive, or sensory functions, and are described according to the type of task and stimuli used during the study, their distribution on the scalp (topography), amplitude, polarity, and latency (Kessler et al., 2016). Early ERP waveforms, occurring 0–200 ms after stimulus onset, are termed *sensory* or *exogenous*, as they are largely modulated by the physical aspects of the presented stimuli (e.g., color, brightness, spatial frequency, complexity). In contrast, ERP waveforms that occur at later time windows after stimulus presentation are termed *endogenous*, as they are determined less by the physical features of the stimulus and more by the reactions of the subject in response to the stimulus or a task. For example, endogenous generated components of P300 and later components (e.g., N400) are sensitive indicators of selective attention and semantic processing in response to a task (Cohen, 2014; Herrmann et al., 2014). As timing is an important factor in the allocation of attentional resources to food stimuli (Wolz et al., 2015), and temporal aspects of this attentional allocation can be measured by ERPs, it is important that ERP measures be employed to improve our knowledge of the differential allocation of attention to food or body shape stimuli in patients with BP-ED. ERP studies are also useful to elucidate potential perceptual, motor, motivational, and emotional impairments linked to BP-ED. A summary of outcome measures of the main ERPs is presented in Table 1.

**Table 1:** ERP components by time latency and functional significance.

**ERP**	**Time latency after stimulus onset (ms)**	**Functional significance**
N100 (or N1)	90–200	Early sensory processing and automatic orienting (Hillyard et al., 1998; Vogel and Luck, 2000; David et al., 2011; Kappenman and Luck, 2011)
Error related negativity (ERN)	80–150	Individual error processing (Gehring et al., 2011, 2018; Wang et al., 2020)
P200 (or P2)	100–250	Early selective attention Attentional and cognitive processing Automatic orienting to stimuli (Crowley and Colrain, 2004; Pacheco et al., 2020)
N200(or N2)	180–300	Conflict detection during the regulation of successful behavior (Donkers and van Boxtel, 2004; Folstein and Van Petten, 2007; Riesel et al., 2017; Heidlmayr et al., 2020)
Early posterior negativity (EPN)	200–300	Valence processing Stimulus arousal Attention allocation (Junghofer et al., 2001; Schupp et al., 2003a,b, 2007; Farkas et al., 2020; Hajcak and Foti, 2020; Lemos et al., 2020)
P300 (or P3)	250–500	Measure of attention independently of behavioral responding Visual attention toward stimuli with personal relevance Emotional salience (positive or negative) Working memory update Motivated attention (Polich, 2007, 2011; Lobo et al., 2014; Hajcak and Foti, 2020)
N400 (or N4)	250–500	Processes indexing access to semantic memory (Kutas and Federmeier, 2011)
Late positive potential (LPP)	300–1,000	Conscious allocation of attention Allocation of motivational or emotion-based attention (Hajcak et al., 2010; Brown et al., 2012; Hajcak and Foti, 2020)
Slow positive wave (SPW)	500-6,000	Categorization and response selection processes (Johnston et al., 1986; daSilva et al., 2016)

Additionally, EEG signals can be decomposed into frequencies (e.g., alpha, beta, theta, delta) to look for condition-related oscillatory patterns (Luck, 2014; Kim and Im, 2018). Neural oscillations and their synchronization may provide useful insights into interneuronal communication and integration of neural information that is processed in distributed brain regions (Kim and Im, 2018). This spectral analysis of the EEG enables digital representation of the aforementioned frequency bands (Andreassi, 2010). Changes in specific bands may be an indicator of increased firing rate within certain cell populations, reflecting different stages of cognitive arousal, cognitive processing, and psychopathology (Klimesch, 1999; Makeig et al., 2004). In summary, frequency analysis can additionally provide helpful information to clarify the relationship between brain functions, cognitive functions, and eating behavior. An overview of frequency bands and associated cognitive states is presented in Table 2.

**Table 2:** Frequency bands and associated cognitive states.

**Frequency**	**Band (Hz)**	**Functional significance**
Delta	1–4	Sleep state Arrhythmic delta connected to problem-solving tasks (Niedermeyer and da Silva, 2004)
Theta	4–8	Wakefulness with state of forced attention Creativity Distractibility Inattention (Niedermeyer and da Silva, 2004)
Alpha	8–13	Relaxed wakefulness Meditation Peacefulness (Niedermeyer and da Silva, 2004)
Beta	13–21	Attentive wakefulness Focus Relaxed thinking (Niedermeyer and da Silva, 2004)

In 2012, Lobera et al. conducted the first-ever review of the cerebral electrophysiology of ED (Jáuregui-Lobera, 2012), focusing on studies which explored EEG findings of sleep/resting states in patients with AN. Only three studies included samples with patients with binge eating (Rodriguez et al., 2007; Tammela et al., 2010; Blechert et al., 2011a). Of these, two used frequency-domain EEG analysis (Rodriguez et al., 2007; Tammela et al., 2010), whereas the third applied time-domain (ERP) analysis (Blechert et al., 2011a). Two findings regarding EEG in BP-ED individuals emerged from this review: obese individuals with binge eating showed greater frontal beta activity when compared to those without binge eating, and individuals with BN exhibited affective priming compared with healthy controls. Another review was conducted in 2015 by Wolz et al. (2015). Although not focused on patients with binge-eating spectrum disorders, it did include two studies of patients presenting episodes of binge eating, one with a BN sample (Blechert et al., 2011b) and another with BED subjects (Svaldi et al., 2010). This review focused on ERP findings and did not include any studies using frequency-domain EEG analysis. More recently, two reviews of electrophysiological findings in ED were published. The first, conducted in 2018, investigated abnormalities in the EEG power spectrum in individuals with BN, BED, and obesity (Blume et al., 2019). Of seven studies included (Léonard et al., 1998; Tammela et al., 2010; Hume et al., 2015a,b; Imperatori et al., 2015; Bauer and Manning, 2016; Wolz et al., 2017), only four assessed BN or BED (Léonard et al., 1998; Tammela et al., 2010; Imperatori et al., 2015; Wolz et al., 2017); the other three evaluated obesity alone (Hume et al., 2015a,b; Bauer and Manning, 2016). The authors found that (1) beta activity on resting-state EEG was elevated in fronto-central regions in individuals with obesity and comorbid BED; and (2) beta activity correlated positively with ED psychopathology at rest and following exposure to food cues.

The most recent review was performed in 2019 and focused on ERP in response to food stimuli among individuals with ED and weight disorders (Chami et al., 2019). The authors found that individuals with binge eating exhibited an enhanced attentional response to food cues compared to healthy controls. They also reported that inhibitory control-related ERP components (N200 and P300a) increased during food-specific exposure but did not differentiate overweight from normal-weight groups.

In summary, the current literature provides preliminary evidence of the potential usefulness of electrophysiological methods to assess cerebral function in patients with ED. However, the inclusion of multiple diagnostic rubrics in the same reviews is a common limitation. As EDs are a heterogeneous group, it is important to understand that they are expected to present with different neurobiological features captured by EEG. We conducted this systematic review to collate and analyze the current evidence on EEG findings (including ERP and frequency analyses), in the more homogeneous spectrum of BP-ED. We also aimed to identify additional limitations of the current research to inform futures directions in the field. We seek to provide an overview of current knowledge regarding the electrophysiology of BP-ED, applying different analyses to gain new insights into the pathophysiological processes underlying sensory, cognitive, and affective deficits as well as clinical symptoms in these disorders.

## Methods

### Literature Search

Two authors (JCH and ACD) independently conducted a systematic search of the PubMed, Embase, and Web of Science databases from inception through July 1, 2020, using the following terms: (“binge-eating disorder” or “bulimia” or “abnormal eating”) and (“electroencephalography” or “electroencephalogram” or “EEG” or “brain waves” or “evoked potentials” or “event-related potential”). No filters for date of publication were applied. The electronic search strategy was supplemented by a handsearch for additional articles in the reference lists of included articles and previous reviews. This review adheres to the PRISMA reporting guidelines (Moher et al., 2009).

### Inclusion and Exclusion Criteria

Original articles that performed an EEG method in individuals with BP-ED were included. Cross-sectional or longitudinal studies were required to describe EEG neurophysiological outcome data. The BP-ED diagnosis should be confirmed by a validated assessment instrument based on an international classification system (DSM or ICD). Case studies, articles evaluating sleep EEG, samples composed of children, and reviews were excluded, although their reference lists were screened for additional articles not retrieved in the initial search.

### Data Extraction

Two authors (JCH and ACD) independently screened and selected the studies based on titles and abstracts. After consensus on the first round of selection, both authors independently reviewed the full text of the selected studies to determine their suitability for inclusion, based on the established selection criteria. Disagreements between the two authors were solved by discussion with each other and with the senior author (JCA) until consensus was reached. One author (JCH) extracted data from each study using a form designed for the purpose of the present review. Data of interest included sample size, diagnosis, stimulus type, task performed, measures, primary outcome, and main findings.

### Quality Assessment

Our quality assessment tool included seven questions from the original Newcastle-Ottawa Quality Assessment Scale adapted for cross sectional studies (Herzog et al., 2013), as well as three additional questions regarding EEG quality: (1) “does the author report specific hypotheses and predictions for the EEG measures in the introduction?,” (2) “was the description of EEG data preprocessing steps clear and detailed?,” and (3) “were the stimuli standardized and paired by characteristics?” Our assessment ranged from 0 (minimum) to 10 (maximum).

## Results

**Figure 1:**
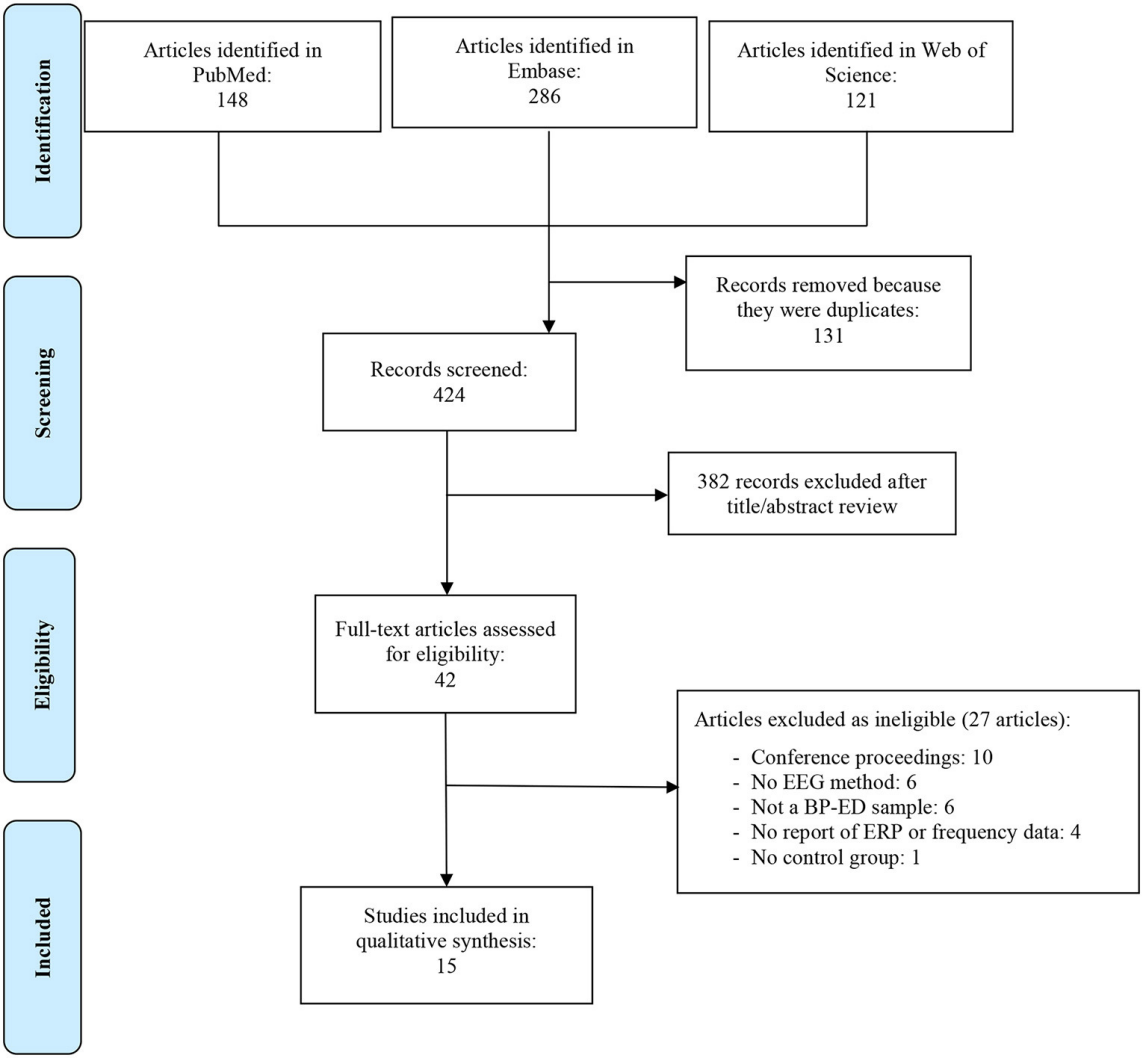
PRISMA flow diagram.

The initial search identified 555 potentially eligible articles. After removal of duplicates, 464 studies remained. Screening of titles and abstracts reduced this to 42 studies, which were assessed for eligibility. After full-text reading, 27 studies were excluded for a variety of reasons (Figure 1). The final sample thus consisted of 15 articles.

Below, we describe the findings of the included articles. Individual sections are provided for time-domain and frequency-domain analyses. It is important to note that although, a few studies included a subsample of participants with AN, we excluded these data from our discussion.

### Time-Domain Analysis (ERP)

We found a total of 11 studies investigating central markers by means of ERP in individuals with BP-EDs (Table 3). These studies employed different stimuli to evoke ERPs in two categories: (1) non-eating disorder-related stimuli, i.e., those not related to ED psychopathology (auditory clicks or facial images, for example); and (2) stimuli related to ED psychopathology (food pictures or body-related images).

**Table 3:** Time domain analysis: studies evaluating ERP waveforms in BP-ED.

**References**	**Sample (*n*)** **diagnostic**	**Stimulus**	**Measures**	**ERP**	**Main findings**
Non-eating disorder related Stimuli					
Otagaki et al. (1998)	AN = 28 BN = 24 HC = 40 (DSM-IV)	Two-tone discrimination task (“oddball” paradigm)	EAT-26; SDS; STAI; BIS-10	P300 250-450 ms	BN group exhibited a prolonged latency compared to HC
Kuehnpast et al. (2012)	BN = 13 HC = 13 (DSM-III/IV)	Neutral, happy, fearful, and angry facial expressions	SCID	N170 120-180 ms	BN group: reduced amplitudes for angry faces compared to HC
				N2 190-260 ms	Higher scores in depression were associated with more negative N200 amplitudes
				P300 270-500 ms	BN group: Higher mean amplitudes compared with HC independent of emotional expression
				P300 300-650 ms	BN group: Higher amplitude for distractor stimuli (P300a) and for targets (P300b)
				SW 350-800 ms	BN group: Increased amplitude and shorter latency compared to HC
Merlotti et al. (2013)	BN = 17 HC = 17 (DSM-IV-TR)	Three-tone auditory oddball paradigm	ERP; SCID-I; BIS-11; EDI-2; BITE; WAIS-R	N2 200-350ms	BN group: Reduced amplitude and shorter latency compared to HC
Blechert et al. (2011a)	AN = 20 BN = 20 HC = 28 (DSM-IV)	Sentences eliciting body shape, weight, and typical eating concerns of ED patients	ERP; EDE; EDE-Q; BDI; RSE; MSES; SCID	N400 340-450ms	BN group: Amplitudes higher (more negative) for incongruent conditions
Food Stimuli					
Svaldi et al. (2010)	BED = 22 GC = 22 (DSM-IV-TR)	Food pictures (high- and low-calorie).	ERP; EDE; DEBQ	LPP 500-800ms	BED group: Increased for high-calorie food pictures compared to GC. No differences between groups on low-calorie food pictures.
				SPW 1000-6000ms	BED group: Larger amplitudes compared to GC. BED group: Larger with high-calorie food pictures compared to GC. No significant group difference in response to low-calorie food pictures.
Blechert et al. (2011b)	BN = 22 AN = 21 HC = 32 (DSM-IV)	Food (high- and low-calorie) vs. non-food related images.	ERP; EDE; EDE-Q; STAI; BDI	EPN 220-310ms	BN group: Higher for food pictures compared with neutral. No difference between high- and low-calorie categories.
Schienle et al. (2017)	EG = 36 (19 BED and 17 BED low frequency) HC = 38 (DSM-5)	Food pictures (high- and low-calorie).Two groups: bitter or water condition	EDI; QADP; BAS	P200 150-200ms	EG: Higher in both food picture categories in bitter condition compared with HC.
				LPP 400-700ms	EG: higher in both food categories in the bitter condition compared with HC.
Wolz et al. (2017)	EG = 19 (BN =12; BED = 7) HC = 20 (DSM-5)	Chocolate odor and pictures	FCCQ; DERS; YFAS; EDI-2; SCL-90-R	N2 180-350ms	EG: higher amplitudes for chocolate pictures primed by chocolate odor compared to neutral odor.
				LPP 300-1000ms	No differences between HC and EG.
Leehr et al. (2018)	OW+BED (*n* = 24) OW–BED (*n* = 23) NWC (*n* = 26) (DSM-IV)	High-calorie food pictures and non-food pictures	BDI-II ASTS	N2 150-250ms	Latencies were significantly larger in OW without BED compared with OW+BED individuals and NWC sample
				ERN 0-150ms	No differences in latencies and amplitudes
Delgado-Rodríguez et al. (2019)	BN = 24 HC = 24 (DSM-5)	Images of high-calorie foods, erotic couples, neutral objects, and unpleasant scenes	EAT; BITE; BDI; FCQ-t; FCQ-s; SHAPS; STAI	EPN 220-310ms	No differences in latencies and amplitudes
				LPP 500-800ms	BN group exhibited a larger amplitude with binge food and erotic cues
Body/shape stimuli					
Mai et al. (2015)	BN = 20 HC = 20 (DSM-IV)	Body-related images (underweight, normal, and overweight)	ERP; SCID-I; RS; STAI; BDI-II; EDI-2	N170 150-220ms	No significant main or interaction effects on amplitude or latency
				P200 180-270ms	Both groups: Lowest amplitudes for underweight body stimuli compared to normal body stimuli BN group: Higher amplitude in response to overweight stimuli compared to normal body stimuli
				N2 250-350ms	Both groups: Most pronounced amplitudes for underweight compared to normal and overweight body stimuli Only HC exhibited more negative N200 in response to underweight compared to normal and overweight body stimuli HC group: Significantly shorter latency compared to BN
				P3 350-450ms	HC group: Higher amplitudes compared to BN to overweight and normal-body stimuli
				SW 500-900ms	More positive for underweight body stimuli compared to overweight and normal. No differences between BN and HC

*ASTS, State Mood Scale; BAS, Behavioral Activation Scale; BDI, Beck Depression Inventory; BES, Binge Eating Scale; BIS-10, Barratt Impulsiveness Scale 10; BIS-11, Barratt Impulsiveness Scale 11; BITE, Bulimic Investigation Test Edinburgh; CG, control group; DEBQ, Dutch Eating Behavior Questionnaire; DERS, Difficulties in Emotion Regulation Scale; EAT, Eating Attitude Test; EAT-26, Eating Attitude Test 26; EDE, Eating Disorder Examination; EDE-Q, Eating Disorder Examination Questionnaire; EDI, Eating Disorder Inventory; EDI-2, Eating Disorder Inventory-2; FCCQ, Food Chocolate-Craving Questionnaire; FCQ-s, Food Craving Questionnaire (state); FCQ-t, Food Craving Questionnaire (traits); HADS, Hospital Anxiety and Depression Scale; HC, healthy control; OW, overweight; MSES, Multidimensional Self Esteem Scale; NR, not reported; QADP, Questionnaire for the Assessment of Disgust Proneness; RS, Restraint Scale; RSE, Rosenberg Self-Esteem Scale; SCID, Structured Clinical Interview for DSM; SCL-90-R, Symptom Checklist-90 Revised; SHAPS, Snaith-Hamilton Pleasure Scale; SDS, Zung's Self-Rating Depression Scale; STAI, State-Trait Anxiety Inventory; TAS, Toronto Alexithymia Scale; TFEQ, Three-Factor Eating Questionnaire; WAIS-R, Wechsler Adult Intelligence Scale – revised; YFAS, Yale Food Addiction Scale*.

#### Non-eating Disorder-Related Stimuli

The first study evaluating ERP in BP-ED individuals was performed in 1997 (Otagaki et al., 1998). The authors hypothesized that, as individuals with ED exhibit some cognitive impairments (McKay et al., 1986; Jones et al., 1991) and the P300 component reflects cognitive function in the brain (Sutton et al., 1965), this ERP could be a psychobiologic marker of eating disordered behaviors. They used a two-tone discrimination task—the oddball paradigm—to assess P300 in 24 BN individuals compared with 40 healthy controls. The oddball paradigm consists of the discrimination of an infrequent sequence or stimulus (the target stimulus) among frequent (non-target) stimuli and is commonly used to assess P300. The BN participants had a significant prolonged P300 latency compared to those in the control group. The authors suggest that this result may reflect that individuals with BN may present a task-related slowing of cognitive processing.

Using another non-eating disorder-related stimulus, Kuehnpast et al. (2012) performed an ERP study in individuals with BP-ED. These authors presented images of facial expressions (neutral, happy, fearful, and angry) to 13 participants with BN and 13 healthy controls. Several ERPs were analyzed: N170, N200, and P300. Compared to healthy controls, the BN group showed (1) a reduced N170 amplitude for angry faces; (2) a higher P300 mean amplitude independent of facial expression; and (3) a higher amplitude for distractor stimuli (P300a) and target stimuli (P300b). These results demonstrated that emotional faces can be processed differently in persons with BN. In conclusion, individuals with BN failed to allocate more attentional resources to motivationally relevant stimuli, which demonstrates that early cortical decoding processes might not be functional. To compensate for this failure, these individuals devote a higher amount of cognitive effort to evaluating facial expressions.

In 2013, Merlotti et al. (2013) conducted another ERP study using a three-tone auditory oddball paradigm in a group of 17 BN participants compared to a healthy control group *(n* = 17). This procedure is a variant of the oddball paradigm which has the possible advantage of allowing assessment of other cognitive processes than those obtained in the traditional paradigm (Wronka et al., 2008). The authors found a reduced amplitude and shorter latency in N200 in the BN group compared to healthy controls. In addition, they reported an association between impulsivity (measured by the Barratt Impulsivity Scale) and the reduction in N200 latency. Authors also reported an increased amplitude and short latency of the target slow wave (SW) and a higher amplitude of the P300 component. Taking together, these observations suggest the occurrence of functional abnormalities in brain activities involved in attention and self-regulatory control (such as a deficit in the inhibitory processes and an excessive allocation of attentive resources to irrelevant stimuli), possibly related to impulsivity in BN.

In summary, the ERP studies using non-eating disorder-related stimuli suggest that individuals with BP-ED may exhibit impaired cognitive processing independently of stimulus characteristics. These alterations may be associated with the attentional deficits observed in this group of individuals.

#### Eating Disorder-Related Stimuli

The first study using food stimuli to assess ERP in BP-ED was performed by Svaldi et al. (2010). The authors compared 22 BED participants with 22 overweight subjects (control group) and assessed late positive potential (LPP) and slow positive wave (SPW) after presenting images of high-calorie and low-calorie foods. The experiment consisted of two sections. In a “viewing section,” participants simply viewed food pictures, while in the “availability section,” participants were instructed to taste some foods during the sequence. An increased amplitude of LPP and SPW for high-calorie food pictures was observed in the BED group compared to controls. Of note, the differences were greater during the “availability section” of the experiment. In conclusion, for women with BED, high-calorie foods may have high motivational properties and attract high levels of attentional resources in information processing.

Blechert et al. (2011b) evaluated EPN in a subsequent study with a sample composed by 22 BN participants and 32 healthy controls. They used a set of high- and low-calorie food pictures, compared with non-food-related images. A higher EPN for both high- and low-calorie food pictures compared to neutral pictures was found in BN group (no difference between high- and low-calorie categories). In healthy controls, EPN were higher only for high-calorie food pictures (with no difference between neutral pictures and those of low-calorie foods). In this study, participants with BN showed a generalized attentional bias for food images, regardless of caloric value. A relevant limitation of this study is that, although the authors used non-food images from the International Affective Picture System (IAPS) as control stimuli, they did not describe the source of the food pictures displayed.

In 2017, Schienle et al. (2017) performed a study using a taste perception paradigm to check if a bitter test (in which the perception of bitterness usually reduces the salience of the food cues presented) could change the reward value of visual food cues. They assessed the ERP components P200 and LPP in a sample of 36 BED participants compared to 38 healthy control individuals. Pictures of high- and low-calorie foods were used to evoke ERP potentials. In this study, the BED group displayed a higher P200 amplitude for both high- and low-calorie food pictures compared to healthy controls in the bitter condition. The BED group also showed higher LPP for both food images during the bitter condition when compared to the control group. Hence, results showed that exposure to a bitter taste did not decrease the late-positive ERP component to visual food cues (reflecting food reward sensitivity) in women with BED. The authors suggested that this atypical bitter response (as bitter taste reduced P200/LPP to food images in healthy women) might be a biological marker of BED and, possibly, related to overeating in these individuals.

Another study was performed by Wolz et al. (2017) in a sample of 12 subjects with BN, 7 subjects with BED, and 20 healthy controls. The authors conducted a clinical experiment using a combination of visual and olfactory chocolate stimuli to assess N200 and LPP. Although chocolate pictures elicited a higher LPP than neutral images, there was no significant difference between the experimental group and controls in N200 and LPP amplitudes. The authors concluded that BP-ED might be associated with lower baseline N200 activity and a higher relative increase in response to chocolate cues than healthy controls, and argued that the lack of significance may have been due to the small sample size or to the olfactory stimulus serving as a confounding factor.

Leehr et al. (2018) performed a study using food-related stimuli in BP-ED individuals to assess N200 and error-related negativity (ERN) in three groups: (I) overweight patients with BED, (II) overweight individuals without BED, and (III) normal-weight controls, using high-calorie food pictures and neutral images. The results showed that N200 latencies were significantly larger in the non-BED compared with BED group. This prolonged N200 latency is related to conflict processing and could be attributed to a compensatory mechanism. Subsequently, the authors concluded that, as the BED group did not exhibit this larger N200 latency, they did not intensify conflict monitoring processes, failing to overcome potential deficits in inhibitory control.

More recently, Delgado-Rodríguez et al. (2019) conducted an ERP study to evaluate the LPP response to food and erotic cues in individuals with BN. The authors hypothesized that as these individuals exhibit aversion to body cues, the motivational significance of erotic images could be increased in women with BN. This study compared the LPP response of 24 women with BN to that of 24 healthy controls. A significant larger LPP amplitude was found in the bulimic group in response to pictures of binge-related foods and erotic pictures. The findings suggest the importance of LPP as an indicator of motivational significance activity of cortical and subcortical areas involved in emotional perception, as LPP is not affected by perceptual characteristics of the stimulus as early ERP components used to be. Thus, the LPP component may be a pathway to assess altered processing food cues and eating disorder-related stimuli in BN.

The first study to investigate electrophysiological parameters in response to body image stimuli in subjects with BP-ED was performed by Mai et al. (2015). In this experiment, the authors assessed the N170, P200, N200, P300, and SW components in a sample of 40 participants (20 with BN and 20 matched healthy controls). The task consisted of the presentation of underweight, normal, and overweight body pictures; participants were instructed to evaluate these images in terms of their reactions (pleasantness and arousal). This study showed that BN subjects exhibited a greater P200 amplitude in response to overweight stimuli compared to normal-body pictures. Regarding N200, healthy controls presented a more negative amplitude in response to underweight body stimuli when compared to normal and overweight body stimuli. In addition, healthy controls exhibited higher P300 amplitudes for overweight and normal-body stimuli compared to the BN group. No differences were found in N170 and SW between groups. Participants with BN also rated overweight body stimuli as bigger and more arousing, highlighting that overweight bodies provoke altered perceptual and cognitive-affective processing. The authors confirmed their hypothesis that individuals with BN exhibit alterations in the processing of female body stimuli in response to underweight, normal-weight, and overweight body images.

Although not using pictures, Blechert et al. (2011a) conducted another ERP study with eating disorder-related stimuli in 2010. In this study, 20 participants with BN and 28 healthy controls were included. The authors used sentences eliciting body shape, weight, and typical eating concerns of eating-disordered patients to assess N400. Overall, when compared to participants with BN, healthy controls showed higher N400 amplitudes (more negative) for incongruent conditions (e.g., “when I gain weight, I feel…accepted”). The results suggested that the neural affective processing of individuals with BN indicated a tighter link between self-evaluation domains and shape/weight concerns as compared to the other groups.

In summary, ERP investigations using eating disorder-related stimuli confirm the findings raised by studies with non-eating disorder-related stimuli in individuals with BP-ED. These subjects displayed a range of impairments from early sensory processing and selective attention through to late cognitive processes, conscious allocation of attention, and allocation of motivational or emotion-based attention.

### Frequency Domain Analysis

A total of five studies focused specifically on frequency-band changes in EEG-measured brain activity in BP-ED samples were retrieved (Table 4). In general, early studies were composed of mixed samples (usually with AN), while more recent studies selected more homogenous samples.

**Table 4:** Frequency domain analysis: studies evaluating EEG oscillatory activity in BP-ED.

**References**	**Sample (*n*) diagnostic**	**Stimulus/Task**	**Measures**	**Frequency band**	**Main findings**	**Comments**
Léonard et al. (1998)	AN = 14 BN = 10 HC = 18 (DSM-IV)	Before meal (resting state) and meal (on starting, during and after the end of the meal)	EDI; HADS; STAI	Alpha, beta, delta, and theta	No significant differences between groups	First study to assess EEG data in patients with eating disorders
Rodriguez et al. (2007)	AN = 16 BN = 12 HC = 30 (DSM IV-TR)	Resting-state EEG (eyes closed)	NR	Alpha 1	HC exhibited increased amplitude compared with BN in temporal, central, parietal, occipital, and limbic sites	Alpha 1 amplitude in central sites was correlated with BMI in patients
				Alpha 2	HC > AN and BN (parietal, occipital, and limbic)	
				Delta and theta	No significant differences between groups	
Tammela et al. (2010)	EG: 12 Obese BE GC: 13 Obese without BE (BES > 26)	Resting-state EEG (eyes-closed) and focused (eyes-open) during food stimuli	BITE; QEWP; TFEQ; DEBQ; BDI	Beta activity	EG presented higher frontal activity in all situations (stimulus-independent)	Entire sample composed of obese individuals
				Alpha, delta, and theta	No significant differences between groups	
Imperatori et al. (2015)	EG: 13 Obese/OW with BED GC: 13 Obese/OW without BED (DSM-IV-TR)	Resting-state EEG (eyes closed)	BES; HADS	Beta	EG: increased lagged phase synchronization in frontal-temporal and occipito-temporo-parietal areas	EEG connectivity values were significantly related to BE, even after controlling for depression symptoms
Wolz et al. (2017)	EG = 19 (BN =12; BED = 7) HC = 20	Chocolate odor and pictures	FCCQ; DERS; YFAS; EDI-2; SCL-90-R	Theta	No difference in frontal power between groups	

*BAS, Behavioral Activation Scale; BDI, Beck Depression Inventory; BES, Binge Eating Scale; BIS-11, Barratt Impulsiveness Scale; BITE, Bulimic Investigation Test Edinburgh; DEBQ, Dutch Eating Behavior Questionnaire; DERS, Difficulties in Emotion Regulation Scale; EDE, Eating Disorder Examination; EDE-Q, Eating Disorder Examination Questionnaire; EDI, Eating Disorder Inventory; EDI-2, Eating Disorder Inventory-2; EG, experimental group; FCCQ, Food Chocolate-Craving Questionnaire; CG, control group; HADS, Hospital Anxiety and Depression Scale; HC, healthy control; OW, overweight; MSES, Multidimensional Self Esteem Scale; NR, not reported; QADP, Questionnaire for the Assessment of Disgust Proneness; RS, Restraint Scale; RSE, Rosenberg Self-Esteem Scale; SCID, Structured Clinical Interview for DSM; SCL-90-R, Symptom Checklist-90 Revised; STAI, State-Trait Anxiety Inventory; TAS, Toronto Alexithymia Scale; TFEQ, Three-Factor Eating Questionnaire; WAIS-R, Wechsler Adult Intelligence Scale – revised; YFAS, Yale Food Addiction Scale*.

The first study assessing frequency-band changes in the EEG in BP-ED populations was performed in 1998 by Léonard et al. (1998). In their study, the sample was composed of 14 participants with AN, 10 with BN, and 18 healthy controls. The authors performed a resting-state EEG followed by a “meal session” in which EEG was recorded before, during, and after a meal. They assessed alpha, beta, delta, and theta frequency bands, but did not find any statistically significant differences between groups.

The second investigation was performed by Rodriguez et al. 9 years later (Rodriguez et al., 2007). The authors evaluated alpha, delta, and theta frequency bands on resting-state EEG in a sample composed of three groups: AN (*n* = 16), BN (*n* = 12), and healthy controls (*n* = 30). They hypothesized that, as EDs might be related to distortion of body image, a topographical evaluation of cortical rhythmicity might reveal EEG abnormalities. In their study, the alpha power amplitude in central, parietal, occipital, limbic, and temporal electrodes was lower in patients with BN compared to healthy controls, especially in posterior regions represented by the temporal, parietal, and occipital lobes. Contradicting their hypothesis, no differences in delta and theta frequencies were found between groups. The authors concluded that participants with BN showed to altered mechanisms of cortical neural synchronization, especially in Rolandic alpha rhythms. As this study included AN and BN samples, is not clear which findings could be related to binge episodes, precluding any conclusions regarding BP-ED neural synchronization.

After these two initial studies, in 2010 Tammela et al. (2010) performed a resting-state EEG investigation using food stimuli in a sample composed of 12 obese participants with binge-eating episodes and 13 obese participants without such episodes. Higher frontal beta activity was found in the experimental group, independently of the stimulus used. The disinhibition factor of the Three-Factor Eating Questionnaire (TFEQ) correlated positively with this increased beta activity. The authors reported no significant differences in amplitudes in alpha, delta, or theta frequencies. The authors proposed that elevated frontal beta activity could be a marker of a dysfunctional disinhibition-inhibition mechanism (Tammela et al., 2010) in those individuals. It is important to point out that the authors did not assess BED categorically; instead, they used the high cutoff point of the Binge Rating Scale (BES≥27) to define a group of binge eaters.

Another EEG frequency-domain analysis study was performed by Imperatori et al. (2015). The authors assessed beta frequency in a resting-state EEG with eyes closed in obese or overweight patients with BED (*n* = 13) and obese or overweight patients without BED (*n* = 13). The EEG functional connectivity showed that the BED group demonstrated an increase in lagged-phase synchronization in the beta frequency in a network involving frontal, temporal, and occipital areas, compared to patients without BED. The authors suggested that this neurophysiological pattern may reflect a hyper-excitability of the frontal control and visual processing networks, which lead BED individuals to be more vulnerable to food cues and exhibit a higher lack of control overeating.

The last study in this section, performed in 2017 by Wolz et al. analyzed theta frequency in 12 BN participants (seven BED and 20 healthy controls) during an event-related brain response experiment to access the influence of olfactory and visual stimuli on craving, inhibitory control, and motivated attention. The authors found no significant difference in frontal theta frequency between groups (Wolz et al., 2017).

#### Quality Appraisal

The 15 included studies scored from 5 to 9 on the modified Newcastle-Ottawa Quality Assessment Scale. Two studies (Rodriguez et al., 2007; Blechert et al., 2011a) reached scores of 5 or 6, nine studies (Léonard et al., 1998; Otagaki et al., 1998; Tammela et al., 2010; Kuehnpast et al., 2012; Merlotti et al., 2013; Mai et al., 2015; Wolz et al., 2017; Leehr et al., 2018) 7 or 8, and four studies (Blechert et al., 2011b; Imperatori et al., 2015; Schienle et al., 2017; Delgado-Rodríguez et al., 2019) scored 9. The major weaknesses found in the studies included (1) absence of sample size calculation, (2) comparability of the groups (e.g., large differences between subgroup characteristics, such as BMI) and (3) the absence of a standardized and paired stimulus used in the tasks.

## Discussion

Our systematic review found a total of 15 studies evaluating electrophysiologic data in individuals with BP-ED. Most articles (11) were ERP studies, while five performed EEG frequency analysis. Taken together, the results of the studies included this review point to a series of electroencephalographic changes in individuals with BP-ED. Regarding time-domain analysis, changes were found at different times after the stimulus onset. Altered initial ERPs (N200, P200, EPN), responsible for early sensory processing, attention allocation, valence processing, and cognitive/attentional processing, demonstrate an altered initial cognitive response, mainly elicited by eating disorder-related cues (food pictures, body images). In addition, changes in late ERPs (LPP and SPW) can reveal an impaired allocation of motivational attribution or emotional-based attention. Thus, these ERP changes may indicate that individuals with BP-ED have a different cognitive processing of stimuli. Despite fewer studies, frequency analysis investigations revealed that individuals with BP-ED exhibit specific alterations in EEG-based brain activity. The increased beta activity, predominantly in fronto-temporal and occipito-temporo-parietal areas, point to a dysfunctional brain network possibly involved in attentional focus toward food stimuli and an increased awareness and vulnerability to food cues.

It bears stressing that our findings are in line with current knowledge of the pathophysiology of BP-ED conditions (Kessler et al., 2016; Steinglass et al., 2019). After an extensive review of the literature, Kessler et al. (2016) proposed a neurobiological basis of BED based on recent neuroimaging, neurocognitive, and genetic data. Their proposal included: (i) altered function in cortical and striatal areas; and (ii) cognitive deficits in executive function, inhibitory control, attention, decision making, and mental flexibility. Another important aspect observed by the authors was the differential response of BP-ED individuals to environmental cues, particularly food-related cues. They proposed that altered cortical function contributes significantly to the altered reward sensitivity and impulsivity observed in BED individuals. Likewise, Steinglass et al. (2019) reviewed the neurobiology of BN and found data suggesting an increased reinforcing value of food, changes in the brain reward system in association with food, and an inhibitory control that is particularly pronounced in the context of food cues.

Some electrophysiological findings observed in the studies included in this review may be related to an overlap with symptoms observed in other mental disorders closely related to BP-EDs. Several other disorders are characterized by impulsivity and behavioral disinhibition, such as ADHD, alcoholism, and other substance use disorders (Imperatori et al., 2015). In these conditions, beta activity and beta coherence have been observed. Abnormal cortical processing along multiple time windows might be related to alterations in oscillatory EEG activity in the beta band frequency along prefrontal-visual cortical pathways. Thus, it is interesting to analyze electrophysiological data from other conditions that are frequently comorbid with ED to help elucidate possible correlates and suggest new directions for cerebral electrophysiology research on this topic. As impulsivity is an important feature of BP-ED, we examined two other conditions associated with impulsivity impairment: substance use disorders (SUD) and attention-deficit/hyperactivity disorder (ADHD). A meta-analysis carried out by Littel et al. (2012) evaluated electrophysiological indices of biased cognitive processing in individuals with SUD. These authors found that P300 and SP (ERPs associated with attentional resources to motivationally relevant stimuli) were significantly larger in SUD participants compared with controls. In addition, SUD individuals exhibited enhanced electrophysiological processing of substance-related cues as compared to neutral cues. This can be interpreted in terms of substance users' motivated attention. In the same line, Johnstone et al. reviewed the ERP literature in ADHD over the last 10 years and found altered N200 and P300 patterns, usually preceded by differences in earlier components (N100 and P200), and in ERN and error positivity (Pe), reflecting problems in selective attention, inhibitory control, and error-processing components. It is interesting to note that several findings presented in these SUD and ADHD studies are quite similar to those found in some BP-EDs, with which they may share some neuropsychological features (Kaisari et al., 2017).

Several limitations of the included studies should be considered. More homogeneous samples in terms of diagnosis and clinical status (illness duration, severity level) are needed. Other parameters, such as gender and clinical comorbidities, can act as confounders and should be addressed as such. In addition, studies usually assessed small samples and did not perform *a priori* sample size calculation, which can limit the external validity of the results. The lack of standardization in EEG paradigms hinders comparison between studies and made it impossible to perform a meta-analysis. Finally, there was a heterogeneity in the stimuli presented and the ERPs measured, with no standardization of the food pictures used across different studies.

Future cerebral electrophysiological studies are warranted in the field of ED, as it appears that EEG findings can provide particularly useful insights into ED neurobiology. We believe that forthcoming research should help clinicians understand whether the EEG changes observed in individuals with BP-ED are state or trait-related. Longitudinal studies could clarify this important aspect. An example of this approach was employed recently in a study conducted by Chami et al. (2020), who piloted a trial of inhibitory training involving participants with BN and BED, exploring behavioral outcomes (i.e., reaction times, omission errors, and commission errors) and changes in N200 and P300 from baseline to post-intervention. Even though participants in the intervention group showed reductions in binge eating frequency, the neural processes underlying this clinical effect could not be entirely uncovered by the post-intervention EEG. The authors concluded that it still unclear whether the null findings reflect an absence of change in neural activity over time or an inability of the measures to detect change; they suggested that future research explore different task parameters.

In conclusion, altered EEG parameters are seen in individuals with BP-ED, suggesting several impairments in neural processing at different levels. Research is still incipient, and future studies need to overcome several shortcomings to confirm the utility of EEG techniques in the assessment of patients with BP-ED. If confirmed as a window to assessment of brain dynamics, EEG should help elucidate the underlying mechanisms of binge eating.

## Data Availability Statement

The original contributions presented in the study are included in the article/supplementary material, further inquiries can be directed to the corresponding author.

## Author Contributions

JH and AD: literature search and manuscript preparation. ID and JA: review of data and manuscript. MD and EV: review of manuscript. All authors contributed to the article and approved the submitted version.

## Conflict of Interest

The authors declare that the research was conducted in the absence of any commercial or financial relationships that could be construed as a potential conflict of interest.

## Abbreviations

AN: Anorexia nervosa
BN: Bulimia Nervosa
BED: Binge eating disorder
BP-ED: Binge-Purge Eating Disorders
ED: Eating disorder
EEG: Electroencephalography
EPN: Early posterior negativity
ERN: Error related negativity
ERP: Event-Related Potentials
LPP: Late positive potential
SPW: Slow positive wave.
